# Family Therapy in Poland: Development and Current Perspectives

**DOI:** 10.1007/s10591-013-9257-3

**Published:** 2013-03-21

**Authors:** Barbara Józefik, Bogdan de Barbaro, Grzegorz Iniewicz, Irena Namysłowska

**Affiliations:** 1 Laboratory of Psychology and Systemic Psychotherapy, Family Therapy Unit, Department of Child and Adolescent Psychiatry, Jagiellonian University Medical College, Cracow, Poland; 2 Family Therapy Department, Department of Psychiatry, Jagiellonian University Medical College, Cracow, Poland; 3 Clinical Unit of Department of Child and Adolescent Psychiatry, University Hospital, Institute of Psychology Jagiellonian University, Cracow, Poland; 4 Department of Child and Adolescent Psychiatry, Institute of Psychiatry and Neurology, Warsaw, Poland

**Keywords:** Family therapy, Poland, History, Current issues

## Abstract

The authors of the present article describe the historical context of family therapy in Poland and current issues in the field. They highlight the fact that Polish therapists first began to develop the field after coming into contact with family therapy leaders from the United States and Western Europe. With the political breakthrough of 1989, there were new opportunities for multilateral cooperation, attendance at international conferences, and the exchange of experiences. Currently, the work of Polish family therapists, the place of family therapy among other forms of psychotherapy, and the related problems and challenges do not differ from other European nations.

## Introduction

This article describes the development and current state of family therapy in Poland.^1^ The first section describes the historical context and is followed by a section that discusses the position and place of family therapy in psychiatry. Subsequent sections include descriptions of organizational development, research, and training issues. In the last sections of the article, the authors focus on the practice and models of family therapy in Poland and the current challenges facing the Polish family therapy community.

## Historical Context

Family therapy in Poland has a relatively long history. The first experiences date back to the 1970s, and three periods can be identified in the four decades that followed. The first period covers the seventies and eighties; the second period covers the time until Poland regained freedom in 1989 and the nineties; and the third period encompasses the current decade of the twenty-first century.

During the first period, family therapy was practiced in the academic centers situated in Krakow and Warsaw and focused on selected families, especially families of adolescents and families with a first-time schizophrenia diagnosis. At the very beginning, therapists based their work on their previous experience, which was mainly psychodynamic, practicing individual or group therapy. Some therapists could also rely on knowledge obtained while studying abroad or completing internships in centers where family therapy had been practiced longer. Gradually, after the professional literature was reviewed, training was completed in foreign centers, and cooperative relationships were developed with Yrjö Olavi Alanen (a Finnish psychiatrist whose study titled *Schizophrenia*—*Its Origins and Need*-*Adapted Treatment* played a significant role in the approach to therapy in Poland), Professor Helm Stierlin (a German psychiatrist, psychoanalyst, and systemic family therapist from Heidelberg University), and other significant figures in the field, the systemic family paradigm was incorporated into the clinical practice of the adolescent unit of the Krakow Psychiatric Department.

It is important to emphasize that the person who introduced the family paradigm and working with families into clinical practice was Maria Orwid, along with her team. Within the framework of child and adolescent psychiatry that she founded, family therapy began to be applied and used in various contexts. In 1983, the Family Therapy Outpatient Unit was established. It was managed by Barbara Józefik and focused on family therapy for children and adolescents. At the same time, family consultations were introduced as a standard procedure in the inpatient adolescent unit, and in 1988, the Home Hospitalization Unit, managed by Ryszard Izdebski, was founded to offer family therapy at patients’ houses.

During the same period, in 1978–1979, Professor Irena Namysłowska, a psychiatrist from Warsaw, was trained in the USA at the Department of Family Therapy at the University of Virginia. She was trained in structural therapy by the American family therapist David Waters, who was a student of Salvatore Minuchin, a founder of the approach who was born in Argentina. After returning to Poland, Professor Namysłowska practiced family therapy at the Department of Psychiatry at the Warsaw Academy of Medicine. Training programs for family therapy were also introduced, organized mainly by the Section of Psychotherapy of the Polish Psychiatric Association. Professor Namysłowska obtained further training in 1985/1986, again in the US in systemic therapy at the Ackerman Institute. This training was made possible with the help of Donald Bloch, a physician, psychiatrist, psychoanalyst, family therapist, and editor of *Family Process and Family Systems Medicine,* who introduced her to the staff of the Institute and allowed her to participate in many seminars and training sessions. Upon returning to Poland, Professor Namysłowska once again introduced state-of-the-art knowledge on systemic therapy to the Department of Psychiatry, along with one of the first one-way mirrors in Poland. Anna Siewierska actively participated in the family therapy training sessions and also trained many family therapists herself.

Concurrently, in another academic center in Warsaw at the Institute of Psychiatry and Neurology, Anna Pohorecka and her co-workers performed family therapy in the newly opened Family Therapy Unit. Family therapy also began to appear in centers not associated with academic healthcare, such as the Synapsis center in Warsaw, where Ryszard Praszkier was the herald of family therapy.

In the second half of the 80s, the systemic family paradigm predominated in the few centers that had introduced family therapy; the approaches most commonly used were the Milan Strategic approach, the structural approach, and the trans-generational approach.

During this period, there were also some important visits from well-known therapists from the USA and Germany who had inspired Polish psychotherapists to practice family therapy (including Lyman Wynne, an American psychiatrist, psychologist, and pioneering family therapist who was a professor at the George Washington University Medical Center; Don Bloch; and Helm Stierlin and his wife, Satu Stierlin, from Heidelberg University). Polish family therapy received substantial support from Western centers. This support was illustrated by the many invitations from other countries: Professor Helm Stierlin invited Kazimierz Pietruszewski, a psychiatrist from the Department of Child and Adolescent Psychiatry, for several months of training in residence; Professor Lyman Wynne of the University of Rochester in New York invited Krakow psychiatrist Bogdan de Barbaro to stay at the local center for a year of training. Upon his return to Krakow, Bogdan de Barbaro established the Family Therapy Department.

The second period in the development of family therapy began in 1989, which was the most significant year in Polish history since the end of the Second World War. The free parliamentary elections and the collapse of communism ignited a process of social and economic change, introducing a parliamentary democracy and a free market in place of the previous socialist system. The transformation changed the context in which many institutions functioned, generating a number of initiatives and new social energy at the same time. There was an increased interest in psychotherapy as a whole and family therapy in particular. This change resulted in greater openness to the West and greater cooperation between academic institutions, as well as greater cooperation within the psychotherapeutic community. Consequently, large-scale training activities began taking place in various Polish cities, encompassing large professional groups consisting mainly of psychologists and medical doctors. At first, eminent foreign family therapists led the trainings Successive visits from Western therapists attracted the interest of a growing community of family therapists. At the beginning of the 90s, there were visits, workshops, lectures, and seminars by the following therapists, among others: Tom Andersen, a professor of social psychiatry at the Institute of Community Medicine, University of Tromso, Norway and the initiator of reflective processes in therapeutic practices; Gianfranco Cecchin, one of the originators of the Milan Systemic School of Family Therapy; Mony Elkaim, the co-founder of the European Family Therapy Association; Klaus G. Deissler, the director of the Marburg Consultation Group and one of the pioneers of systemic and postmodern forms of consultation and therapy in Germany; Maurizio Andolfi, a professor of psychology at La Sapienza (University of Rome) and the director of the Accademia di Psicoterapia Familiare in Rome, Italy; Gill Gorell Barnes, a senior lecturer at the London Tavistock Clinic; Alan Cooklin, an honorary senior lecturer at University College London; Eia Asen, a consultant child psychiatrist and psychotherapist who has been the director at Marlborough Family Service for many years and a consultant psychotherapist at the Maudsley Hospital; Hugh Jenkins, a psychotherapist and a member of the Institute of Family Therapy in London; Florence Kaslow, a visiting professor of psychology at the Florida Institute of Technology; Manfred Cierpka, a professor of psychosomatic and family therapy at the Göttingen University; and Michael Wirshing, the medical director and chairman of the Department of Psychosomatic Medicine and Psychotherapy at Albert-Ludwigs-University in Freiburg, Germany. It is important to emphasize the exceptional importance of the cooperation with the Institute of Family Therapy (IFT) in London, which enabled IFT therapists to conduct workshops regularly in Poland and Polish therapists to visit institutions in London that practiced family therapy. This exchange of experiences was extremely inspiring, especially for those who were entering the field. Cooperation with Klaus Deissler, who came each year for several years for consultations on a reflecting team model, was substantial and important.

In June of 1989, Satu Stierlin from Heidelberg was invited to run the first group on the family of origin of the therapist. This very important event encouraged Polish family therapists to use this method. After October of 1989, two other groups were formed, and since then, genogram work has been included in routine training for therapists who practice family therapy.

Since 1989, family therapy has been used as the main method for treating children and adolescents in the Department of Child and Adolescent Psychiatry at the Institute of Psychiatry and Neurology in Warsaw. The systemic training used live supervision and the one-way mirror. At the same time, family therapy has also become the main treatment paradigm in some outpatient units for emotionally and mentally ill patients.

The cooperation with the international family therapy community proved beneficial when Maria Orwid, who was a well-known Polish family therapist at the time, participated in founding the International Family Therapy Association in 1987 (and became a member of the first board of directors of the IFTA) and later cooperated with the European Family Therapy Association, which eventually developed a formal relationship with the National Family Therapy Organizations of the EFTA.

In 1990, an important event took place that many perceived as crucial for the development of family therapy in Poland. In cooperation with the IFTA, Polish therapists organized an international conference in Krakow: *Family Therapy*—*The Context We Live in*. Many recognized the conference as a significant cultural and scientific event, and approximately 750 family therapists participated. The conference created a unique opportunity for the mutual exchange of experiences and added to the increasing popularity of family therapy and systemic thinking.

In the mid-90s, family therapy was spreading rapidly outside academic centers. Those who completed systemic family therapy training courses began to introduce the methods into their own practice, mainly in psychological and psychiatric counseling. At that time, a growing interest in family therapy was observed among professionals and non-professionals.

In recent years, narrative ideas, object relation theories, attachment theories and feminist ideas have been incorporated into family therapy practice (Józefik and de Barbaro 2004; Józefik and Iniewicz 2008; Tryjarska 2010). The constructionist-narrative paradigm is increasingly affecting the thinking of family therapists (Chrzastowski and de Barbaro 2011; Górniak and Józefik 2003). Currently, therapeutic relationships in the process of family therapy and the family therapist as a person are points of special interest. Among systemic family therapists, couples therapy has been increasingly appealing for several reasons (the transformation of Polish families in response to the pronounced socio-economical-cultural changes in Poland, the changes in the roles and positions of women and men within marriage, and the growing number of divorces) but mostly because of the belief that couples’ relationships are very important and should be improved and saved if possible. Couples therapy is practiced by psychotherapists of various theoretical orientations (quite often by those who combine psychodynamic and systemic approaches), and based on our knowledge, it is practiced in private outpatient centers more often than family therapy. Treatment centers often advertise that they offer family therapy, which is mostly couples therapy in practice.

## Family Therapy and Psychiatry

When analyzing the historical context of the development of family therapy in Poland, it is worth underlining the close relationship between family therapy, psychiatry, and psychotherapy. The people who introduced and developed family therapy in Poland made significant achievements in both of these fields, and they discovered family therapy as yet another field of interest. Thus, it was possible to introduce systemic thinking into psychiatry, graduate and postgraduate courses, and diagnostic processes and treatment. Consequently, family therapy was introduced as a standard procedure for treating many disorders, especially in children and adolescents (de Barbaro and Namysłowska 2011; Józefik 2004). Historically, some family therapists started their practice working with children and adolescents suffering from various psychic disorders. Other therapists worked with adult patients suffering from schizophrenia (de Barbaro 1999). Thus, Polish therapists gathered rich and diverse experiences. However, it seems that the interplay between family therapy and psychiatry created both advantages and disadvantages. The obvious advantages included the application of the systems approach to the family context, both in the diagnosis and in the understanding of patients’ problems. For children and adolescents, this approach was reflected in the interest shown in the interplay between a patient, his/her family system, school and peer communities, etc. Systems-based methods also allow for the integration of various approaches: medical, psychological, therapeutic, and pedagogical.

Family therapists working with adult patients suffering from schizophrenia must consider both the specific character of the condition and the phase of family development among their patients (de Barbaro 1997). Consequently, family therapy has a crucial role to play in combination with the psycho-educational approach, which stemmed from research on the actor of emotional expression. Other components of this approach include educational programs explaining schizophrenia, training sessions in communication and problem solving, etc. Family therapy or family consultation sessions have also become a permanent feature of the work in many clinical wards. In addition to these advantages, such programs prepare a family for the possibility of future therapy conducted on an outpatient basis after the patient’s discharge from the hospital.

However, the relationship between family therapy and psychiatry also has a negative aspect—patients are referred to therapy by psychiatric hospital wards. Some patients and their families view this experience traumatically because of social stigma, which may negatively influence the onset of therapy and the potential for stable contact between a family and a patient. Very frequently, families are inclined to shrug off the burden related to the psychiatric treatment of their members. Many stereotypes about the treatment in psychiatric wards are still present in Poland. In practice, these stereotypes result in the tendency to conceal the use of therapy services, even from more distant relatives. Another problem concerns the understanding of psychotherapeutic treatment by patients themselves. Medical services are usually viewed as visits to a specialist who prescribes appropriate medicines. This attitude may sustain the medical model of illness and therapy. Nonetheless, a majority of families participate in family therapy and are encouraged to reflect on the essence of illness and treatment. Thus, therapists reinforce families’ conviction that common conversations about the most painful issues are not only possible but also very helpful.

## Organization

In 1998, the increasing interest in family therapy was reflected in the formation of the Family Therapy Scientific Section (FTSS) within the Polish Psychiatric Association (PPA). Its founders were members of the Psychotherapy Scientific Section of PPA who believed that the rapidly developing field of family therapy should have its own representation. The first president of the section was Professor Maria Orwid.

The decision not to establish a separate Family Therapy Society was based on political grounds. As mentioned above, psychotherapy has been closely connected to psychiatry in Poland. It seemed beneficial to remain within the structure of the large association as numerous changes occurred: changes in the Polish National Health Service that had been occurring since 1999, the introduction of a new reimbursement system for medical treatment costs, and the changing regulations on the psychotherapeutic practices of psychologists. This decision led to a number of positive outcomes. First, the section cooperates very closely with the Psychotherapeutic Section of the Polish Psychiatric Association, one of the largest Polish psychotherapeutic associations, which has introduced standards for psychotherapeutic training in Poland, criteria for assessing training programs for psychotherapists and psychotherapy supervisors, and regulations for conducting the examination that align with the directives of the European Association for Psychotherapy. Because of this cooperation, all decisions related to the issues discussed above are made during joint meetings of the managing boards of the two sections. This activity is extremely important because there is currently a regulation on the professions of psychology and psychotherapy being developed in the Ministry of Health.Polish law does not regulate many issues related to psychotherapy, and therefore, the procedures introduced by the two sections have been used as the basis for regulations concerning psychotherapy and family therapy for many years. Moreover, both boards cooperate closely with the Psychotherapy Section of the Polish Psychological Association to unify the standards and curricula for psychotherapeutic trainings and courses.

The FTSS is a national organization that represents family therapists in the National Family Therapy Organizations of the European Family Therapy Association (NFTO EFTA). Today, FTSS has 356 members and holds annual national conferences devoted to selected issues.^2^

Moreover, there is another association for family therapists in Poland: the Wielkopolskie Systemic Therapy Association. It closely cooperates with family therapists from Heidelberg and organizes training in family therapy. It is a regional association that is connected to the academic center in the city of Poznan.

## Research

It is worth emphasizing that the practical implementation of family therapy in Poland was preceded by an interest in the theory of families and research. The first Polish research on family relations was conducted in the mid-70s. Research into many different aspects of family functioning is still an important field of interest for many scientists. The research addresses questions about varied topics, such as marital relations, family relations, intergenerational patterns for various psychological disorders, transgenerational patterns of trauma, somatic illnesses, and crisis situations. Research is conducted by family therapists who also act as lecturers and as academic teachers and by theoreticians. Recently, research has become more and more focused on the psychotherapeutic process in family therapy.

Family therapists are authors of numerous publications: books and handbooks have helped to popularize this field in Poland (de Barbaro 1994; Namysłowska 2000; Orwid et al. 1991). Some of them are mentioned in this text. It is not insignificant that the current psychiatry textbook for medicine students has a few pages devoted to basic information about family therapy, and a textbook for both adult and child/adolescent psychiatrists offers an entire chapter on the subject (de Barbaro and Namysłowska 2011; Józefik 2004).

## Education and Training

As mentioned above, family therapy in Poland was primarily developed in university clinical centers. It is also in these centers that most of the trainings in family therapy are held. The systemic thinking paradigm and family therapy are introduced at several levels of education. Basic information is provided to students in psychology and medicine departments in courses that are part of the regular curriculum. More advanced knowledge is offered during specialty internships. Furthermore, the training for the psychotherapist certificate includes family therapy as a very significant module. As mentioned earlier, there is still no legal regulation of the psychotherapist profession, and therefore, psychotherapy training is regulated by both sections of PTP, and training in family therapy and systemic understanding of family relations is governed by the Family Therapy Scientific Section (FTSS). The latter training program is usually a 3-year (370–420 h) program that includes theory, psychotherapeutic skill exercises, genogram work on the therapist’s family of origin, and supervised practice These programs are designed and intended for certified psychotherapists or people who want to broaden their systemic and practical skills and work in psychiatric and psychological institutions for children and adolescents or in the social welfare system. Individuals who complete the program do not receive a family therapist certificate, but they do receive confirmation that they have finished the course. The psychotherapist certificate is reserved for individuals who successfully pass the examination after completing a 4.5-year accredited training program with no less than 1,200 program hours that also includes other psychotherapeutic approaches as well as elements of psychiatry and psychology and after meeting some formal requirements. Among the programs that prepare individuals to receive a psychotherapist certificate that are accredited by both sections of PTP, 3 are basically systemic because the systemic unit constitutes the largest part of these programs. It should also be emphasized that the National Health Fund (NFZ) recognizes the value of these programs; for the last 2 years, it has paid more for psychotherapeutic services offered by those who have received training under supervision. To enter the program, participants are required to have completed university-level education in the field of psychiatry, medicine, pedagogy or another field of social and human studies. The training costs are paid by the participants and amount to 800–1,000 EUR annually, depending on the academic center.

Despite the popularity of the systems-based approach to family therapy, the trainees who work in locations other than academic centers often lack appropriate support and regular supervision in their subsequent daily psychotherapeutic work. A solution to this problem may be to increase the number of specialists outside of academic centers and to increase the cooperation between these units and university clinics.

In family therapy courses, couples therapy is only briefly addressed, and there are only a few separate courses on marital therapy, none of which are provided by the Family Therapy Section of PPA. This situation may change in the near future as many psychotherapists declare a need for training in couples therapy.

## The Practice of Family Therapy

The fact that family therapy has become a standard procedure in treatment represents a significant achievement of family therapists. Consequently, family therapy is reimbursed by the National Health Fund (the main insurance company in Poland). However, as previously mentioned, for the last 2 years, family therapy has been undervalued and not priced appropriately. The recognition of family therapy as a basic treatment for emotional and psychiatric problems in children and adolescents is a significant accomplishment of the Polish therapeutic community. However, this does not mean that other forms of therapy are excluded. Broad, systemic and contextual thinking allows for the integration of different types of therapy. Family therapy is used in inpatient care, in outpatient units, and also in private practice. There are family therapy teams in every psychiatric department for children and adolescents within university centers in Poland. All of them have a one-way mirror, which allows for teamwork and live supervision. Systemic family therapy is considered one of the main methods for treating children and adolescents suffering from adolescent crises, depression, or conduct disorders, and it is an important part of the treatment plan for other children and adolescents, such as girls suffering from anorexia and psychotic adolescents. Parental training is considered a very important part of the treatment for children with ADHD and conduct disorders.

A different, more complicated situation exists in adult psychiatry. In some (unfortunately few) departments of psychiatry, family therapy is central to the treatment plan for persons suffering from mental disorders. At numerous other psychiatric wards, the family paradigm is not an important part of the treatment plan, and the family is only offered psycho-education. However, one may say that family is important to the success of treatment and represents an important third point in the triangle: patient—treating institution (represented by the physician)—family. As far as social services are concerned, family therapy is a well-developed practice in social services for children and adolescents. The growing interest in the systemic approach, and especially in systemic consultation, can be observed within the education system. This interest results from the fact that the former model used by psychologists and pedagogues employed in the education system has proven ineffective in dealing with school, family, and other systemic problems. Many staff members of the Psychological and Pedagogical Counseling Centers (Poradnie Psychologiczno-Pedagogiczne) who work in the Ministry of Education received training in family therapy. It is worth emphasizing that some of those centers changed their structure and became psychotherapeutic institutions offering, among other services, family therapy. Parental skills training is offered to parents with children with conduct disorders and children suffering from ADHD; systemic therapy is also offered to other children. Family therapy for adults is available and offered mainly in rehabilitation centers.

In 2008, there was an attempt to describe the institutional context for family therapy practice in Poland. To accomplish this goal, 396 questionnaires were sent to psychiatric, psychotherapeutic, and psychological institutions, as well as to individuals. The survey concerned, among other things, specialized education in family therapy, obtaining a psychotherapist certificate, the availability of regular supervision, approaches used, cooperation with other professionals, and the types of problems presented by clients (Józefik and Maryon 2008). In the end, 40 responses were received from the institutions. In 31 of them, family therapy was free of charge for clients: 25 were financed by the municipality, 5 were financed through social services, and 1 was financed by a non-profit foundation. The other 9 institutions offered family therapy for a fee. In the organizations that sent responses, therapists worked in teams of 2–12 people, with 5–8 members on average. There were a total of 185 therapists conducting family therapy. In addition to family therapy, the centers also used individual and group therapy and offered therapeutic-educational groups and support groups, as well as family mediations and social, legal, and psychological counseling. Of the employees, 36 % held a psychotherapist certificate, and another 33 % were participating in the training program and preparing for the certificate examination. The majority of the individuals working with families had completed special training in systemic family therapy. It must be noted that private psychotherapeutic practice has developed significantly in recent years in Poland. The field includes both experienced, older psychotherapists and practitioners at the beginning of their professional careers. Young psychotherapists (the 3^rd^ generation) actively develop and expand their skills by attending conferences and training workshops. The majority of psychotherapists who offer psychotherapy in private practice and also hold a part-time job at a national institution usually prefer individual therapy and couples therapy. Family therapy, on the other hand, is typically practiced in institutional settings, which might be desirable because regular supervision is possible and support can be easily accessed in situations of impasse.

It is also important to note that the Polish Catholic Church has its own network of counseling centers that help families in crisis through family counseling and family therapy. The psychologists and psychotherapists employed there adhere to the rules of the Roman Catholic philosophy.

## Preferred Models of Family Therapy

It is not easy to say which theoretical approach is dominant. Systemic family therapists employ a variety of approaches, such as the contextual approach, the Milan school, the structural approach, and the trans-generational approach. To an increasingly large extent, they modify their ways of thinking and therapeutic techniques using approaches based on social constructivism. As mentioned previously, in the recent years, an approach based on the constructionist-narrative paradigm has become increasingly popular. For many therapists, the narrative approach (mainly Michael White and David Epson’s approach) is particularly important, as is the model based on Tom Andersen’s reflecting team. Lately, there has been significant interest in the dialogical approach in family therapy.

The models of therapy applied depend on the reported problems. The majority of therapists working with couples use object-relation theory or attachment theory, and some work within a psychodynamic frame of reference. Those working with psychotic patients are more eclectic; they often use psycho-education but also use a systemic approach.

Currently, it seems that family therapy is at a stage where it does not emphasize its separateness but rather focuses on the elements that it shares with other psychotherapeutic approaches while simultaneously preserving its own specific characteristics. This process is aptly illustrated by the topics discussed at the conferences organized by the Family Therapy Section, which include psychoanalytical and systemic family therapy, ethical issues in psychotherapy (including family therapy), the problem of narration in psychological theories, and the methodology of family therapy.

## Current Issues

There are also important problems in the development of family therapy in Poland. One of the challenges is the lack of statutory regulations regarding the profession of psychotherapy and thus psychotherapy involving families. Given the intensive work by the community, it is hopeful that this problem will be solved by the Polish parliament in the very near future. Another essential issue that the therapeutic community faces is guaranteeing supervision for individuals working in small centers far from training institutions. Earning a supervisor certificate is a long and complicated process, and therefore, meeting all the requirements is easier in large cities. Consequently, outside of areas where it is easy to access supervisors, there are large regions that lack the ability to provide regular, inexpensive supervision.

The aforementioned underpricing of family and couples therapy services by the National Health Fund is yet another issue. Although it is true that the National Health Fund respects and reimburses the services provided by family therapists for the treatment of mental disorders, in the last 2 years, these services have been undervalued. In an environment where institutions must follow strict budgets, the current policy may limit the number of contracted services for family therapy.

In conclusion, one important task for family therapists is ensuring a high level of therapeutic training and practice, and another important task is improving the position of family therapy in therapeutic treatment. The constantly changing socio-economical context forces therapists to be constantly active and to undertake new enterprises to an even greater extent than in the past; however, these activities are now more likely to be related to political issues rather than to psychotherapy and family therapy.

## Footnotes

1 The subject matter was already the focus of two earlier studies: Orwid and Józefik (1997), Józefik (2005). The present article utilizes fragments of the studies mentioned above.

2 The most recent conference, which took place in October 2012, was devoted to the psychotherapist as a person and to the psychotherapeutic relationship. In May 2013, Professors Peter Fonagy and Eia Asen will visit Krakow and conduct a workshop, "Mentalization-Based Therapy with Children and Families".

## References

[CR1] Chrząstowski Sz, de Barbaro B (2011). Postmodernistyczne inspiracje w psychoterapii.

[CR2] de Barbaro B (1994). Wprowadzenie do systemowego rozumienia rodziny. Introduction into systemic understanding of family.

[CR3] de Barbaro B (1997). Pacjent w swojej rodzinie. Patient in family.

[CR4] de Barbaro B (1999). Schizofrenia w rodzinie. Schizophrenia in family.

[CR5] de Barbaro B, Namysłowska I, Bilikiewicz A, Pużyński S, Rybakowski J, Wciórka J (2011). Terapia rodzin. Family therapy. Psychiatria, t. III.

[CR6] Górniak L, Józefik B (2003). Ewolucja myślenia systemowego w terapii rodzin. Od metafory cybernetycznej do dialogu i narracji. Evolution of systemic thinking in family therapy. From cybernetic metaphor to dialog and narration.

[CR7] Józefik B, Namysłowska I (2004). Terapia rodzin. Family therapy. Psychiatria dzieci i młodzieży. Children and adolescents psychiatry.

[CR8] Józefik B (2005). Family therapy in Poland. Context, European Issue II.

[CR9] Józefik B, de Barbaro B (2004). Terapia rodzin a perspektywa feministyczna. Family therapy and feminist perspective.

[CR10] Józefik B, Iniewicz G (2008). Koncepcja Przywiązania: Od teorii do praktyki klinicznej. Attachment theory. From theory to clinical practice.

[CR11] Józefik, B.,& Maryon, M. (2008). *Praktyka terapii rodzin w Polsce a.d. 2008: próba raportu. The practice of family therapy in Poland: 2008 report*. Coroczna Konferencja 3 Sekcji PTP, 17-19 październik, Abstract book (pp. 25–26). Warszawa.

[CR12] Namysłowska I (2000). Terapia rodzin. Family therapy.

[CR13] Orwid M, Józefik B (1997). Die Etwicklung der Fammilientherapie in Polen. Zeitschrift für Systemische Therapie.

[CR14] Orwid M, Józefik B, Pilecki M, Lask J, Dallos R, Kurimay T, Etenyi Z (1991). Training, supervision, consultation. Distance education in family therapy, counselling and supervision.

[CR15] Tryjarska B (2010). Bliskość w rodzinie. Closeness in family relations.

